# The need to reorganize health research systems in pandemic crisis: A prospective study

**DOI:** 10.1002/hsr2.1146

**Published:** 2023-03-13

**Authors:** Erfan Kharazmi, Sedigheh Ostovar, Milad Ahmadi Marzaleh

**Affiliations:** ^1^ Department of Healthcare Services Management, Health Human Resources Research Center, School of Health Management and Information Sciences Shiraz University of Medical Sciences Shiraz Iran; ^2^ Shiraz University of Medical Sciences Shiraz Iran; ^3^ Department of Health in Disasters and Emergencies, School of Health Management and Information Sciences Shiraz University of Medical Sciences Shiraz Iran

**Keywords:** COVID‐19, health system, need assessment, pandemic, research system

## Abstract

**Background and Aims:**

A pandemic has posed a major challenge to health systems all over the world. All countries have realized that the only way to get real growth and development and solve their problems is to use what they have learned from research.

**Methods:**

A descriptive and analytic type of study was conducted with the help of experts in the field of health research. The components affecting the research system were obtained via process approach and content analysis methods, and then the position of each component was identified by the Mic Mac technique.

**Results:**

Seventeen influential structural components in the health research system were identified. The leadership and management components had the most direct and indirect influence among other components. The health promotion component had a greater dependency than the other components.

**Conclusion:**

All health systems need to provide adequate financial resources and manpower to provide a useful research system. Human resources are the most important inputs to such a system. Components such as the research process, research sustainability, quality, or innovation in research can play a balancing role. Having the right infrastructures for creating, transferring, developing, and getting access to knowledge makes it possible to do systematic science. It is hoped that this science will be used in other results of the health research system, like improving the effectiveness or promoting health.

**Abbreviations**
MDI: Matrix of Direct Influences
MII: Matrix of Indirect Influences
MPDI: Matrix of Potential Direct Influences
MPII: Matrix of Potential Indirect Influences

## 1 INTRODUCTION

A pandemic has posed a major challenge to health systems around the world.^1^ Current prevention and treatment methods that have been effective for other infectious diseases cannot be effective enough for this epidemic, and health systems are forced to discover new ways to prevent and treat the epidemic.^2^ One of the most obvious indicators of the growth and development of a country is its technological capabilities and scientific research, so any action to clarify the status of research and the obstacles facing it is important.^3^ Conducting research studies and producing science are crucial in today's world, such that the number of articles submitted, the number of researchers, and the volume of investment in the research sector are considered the main indicators of development in evaluating the scientific record of a country.^4^ All countries have realized that they have no choice but to invest in and use research findings to achieve real growth and development and solve social, health, and economic problems; without research and using its results, they may not achieve sustainable development in its true sense.^5^ The research sector has attracted the attention of individuals in charge of health systems for various reasons. This is because the results of the health research sector are very effective in ensuring and promoting good health for a society, and ignoring them could put people's lives at risk.^6^

Numerous studies on health systems show that different research systems have been formed in most countries of the world^7^, ^8^, ^9^; however, most of these research systems are not effective enough for health systems for various reasons, one of which is the inconsistency and divergence of research in many research systems.^10^, ^11^ Another reason is the lack of a general and coordinated policy for conducting research in these systems.^12^, ^13^ In many cases, research systems may not be effective due to a lack of financial resources or specialized human resources.^14^, ^15^

The so‐called reasons show how important it is for every health system in the world to have an integrated research system.

In Iran, after the emergence of the Corona crisis, there was an urgent need to review the research system, and the managers and professors of the universities of the Health Ministry have tried to design a suitable system through several meetings and sessions. Universities of medical sciences in Iran have similar organizational structures, and there is no difference between universities in different provinces of Iran. Each university of medical sciences, in addition to providing health services, also provides educational and research services. Due to the similar organizational structure of Iranian universities of medical sciences, the results of this study can be generalized to the whole country of Iran. The present study set out to design an integrated pattern for the health research systems and use appropriate quantitative and qualitative methods.

## 2 METHODS

A descriptive and analytic study was conducted; using the opinions of experts in the field of health research, the components affecting the research system were identified, and then the position of each component in the whole system was identified by Mic Mac analysis.

### 2.1 Participants

The statistical population of this study consisted of administrators and professors of Iranian Medical Universities. Reviewing the studies, an initial list of components was prepared to identify the proper components, and this list was then provided to experts. In semistructured interviews, the collective views of 30 key and expert people in the field of health research were extracted, including the heads and deputies of medical sciences universities, the deans and deputies of the faculties of these universities, and professors of health services' management and health information management. The so‐called individuals were selected based on a purposive sampling method and the following criteria:

- Having at least 5 years of executive experience in health research management
- Publication of at least 10 articles in first index journals in which they were the corresponding author
- Membership in one of the research centers of medical sciences universities
- Having a specialized doctoral degree in one of the specialized fields of the medical sciences universities

Individuals who were reluctant to participate in the study or whose access was difficult due to distance or a busy schedule were excluded from the study and replaced by new individuals. Sampling of experts continued until the theoretical saturation of the data was reached, so 30 experts participated in this study (Table 1).

**Table 1:** Demographic characteristics of the participants in the study.

Variable	Number (%)
Age range	
40–50	12 (40)
51–60	12 (40)
61–70	6 (20)
Sex	
Woman	3 (10)
Man	27 (90)
Marital status	
Married	30 (100)
Single	0 (0)
Management experience	
5–10	6 (20)
11–15	14 (46)
16–20	5 (17)
21–25	5 (17)
Level of education	
PhD	17 (57)
MD	13 (43)
Scientific rank	
Assistant professor	8 (27)
Associate professor	13 (43)
Full professor	9 (30)

### 2.2 Data collection

The final list of components of the health research system was obtained using the process approach and content analysis method. In this step, the six‐step Clarke and Braun method was used, including familiarity with the data, creating initial codes, searching for themes, forming subthemes, defining and naming the main themes, and preparing the final list. Expert opinions were identified at three levels of conceptual, structural, and basic components. Naming structural components were done specifically based on the function of these components. Because the primary goal of this study was to develop an integrated model of the health research system during the pandemic crisis, the third‐level (basic) components were excluded from the analysis, and only the first‐ and second‐level components were used to develop the final model. Third‐level components can be used in the operational plans of the research system. For final review and validation, the list of components was given to two scientific experts who were not part of the research sample. Based on their opinions, any changes that needed to be made were made.

### 2.3 Data analysis

After identifying and classifying the effective components in the health research system, Mic Mac analysis was used to determine the position, influence, effectiveness, and stability of the designed system. The identified components were placed in the *n* × *n* matrix, and the variables' effectiveness or influence was scored 0–3 by the experts. Mick Mac matrices were made in four different ways, and then the outputs of the matrices were sorted and analyzed using Mic Mac graphs and maps:

### 2.4 Matrix of Direct Influences (MDI)

(1) Matrix of Indirect Influences (MII)
(2) Matrix of Potential Direct Influences (MPDI)
(3) Matrix of Potential Indirect Influences (MPII)

## 3 RESULTS

The demographic characteristics of the study participants are shown in Table 1.

In this study, 17 influential structural components in the health research system were identified. Table 2 shows the categories and titles of these components.

**Table 2:** First‐ and second‐level components of the health research system in pandemic crisis.

First‐level: conceptual components—title	Second‐level: structural components—title	Third‐level: basic components—frequency
Enhancers	Leadership and management	5
	Hhuman resources	7
	Information systems and databases	8
	Financing	5
	Research process	5
Primary outputs	Knowledge management and science production	8
	Efficiency	2
	Quality	7
	Sustainability	3
	Innovation	4
	Satisfaction	4
	Decision making	6
Final outputs	Responsiveness to community needs	7
	Health promotion	5
	Culturalization	4
	Equity	5
	Effectiveness	3

Although the third‐level components were not used in modeling, for more information, the frequency of these components is listed in Table 3.

**Table 3:** Influence/dependence matrix of health research system factors in pandemics.

No.	Label	Direct influence	Direct dependence	Indirect influence	Indirect dependence
1	Leadership and management	1420	170	2612	158
2	Information systems and databases	1306	397	2307	206
3	Human resources	909	397	899	317
4	Financing	909	170	1861	101
5	Knowledge management and science producing	909	795	771	512
6	Research process	795	454	1394	281
7	Quality	738	568	3	379
8	Sustainability	681	568	0	399
9	Satisfaction	625	795	1	591
10	Decision making	568	625	1	395
11	Efficiency	511	738	0	457
12	Innovation	397	227	144	202
13	Responsiveness to community needs	113	965	0	1301
14	Equity	56	852	0	1268
15	Effectiveness	56	909	0	1367
16	Health promotion	0	1022	0	1578
17	Culturalization	0	340	0	481

With the entry of the second‐level components into Mic Mac, the position of each component in relation to other components and also their role were determined. The leadership and management components have the most direct and indirect influence among other components. The health promotion component has a greater dependence than the other components.

**Figure 1:**
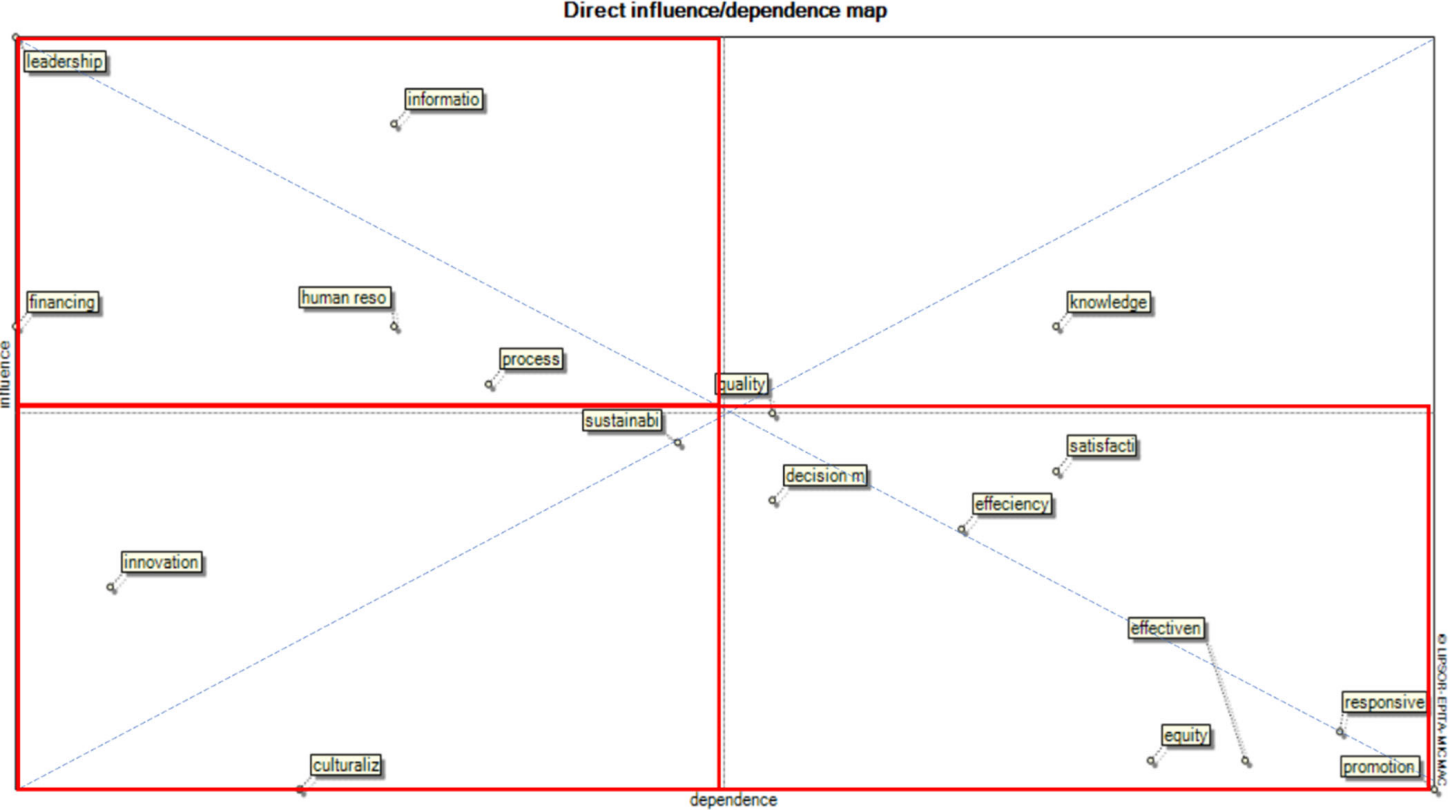
Map of direct and indirect influence of effective components on the integrated health research system.

Management and leadership, systems and databases, financing, human resources, and research process components were identified as input components in the health research system. Innovation, sustainability, and culturalization components are located in the position of independent components of the research system. Knowledge management and science production are the linkage components. Other components act as outputs for the research system. Figure 1 shows the status and position of the components relative to each other. The system under review is stable as only one component is located in the first quarter of the map.

Drawing graphs of the influence of the components in both direct and indirect modes shows the intensity of the relationships between the studied components. In the direct mode, there are strong relationships between the components, while in the indirect mode, a strong relationship can only be observed between the components of management and leadership and health promotion.

## 4 DISCUSSION

Pandemics have had profoundly short‐term and long‐term effects on all aspects of health systems.^16^, ^17^, ^18^, ^19^ Meanwhile, research systems have also been affected by the coronavirus epidemic.^20^, ^21^ In many cases, most resources and facilities of health systems have focused on the treatment sector, and other sectors, such as research, have encountered shortcomings due to the criticality of this epidemic.^22^, ^23^ However, due to the nature and scientific background of the medical profession, from the very beginning of the COVID‐19 crisis, various countries have tried to fight against this epidemic by paying attention to research activities, especially in the field of vaccine production.^24^, ^25^ In this study, professors and administrators interested in research examined various aspects of this epidemic during various sessions and tried to keep this part of the health system active and effective by creating a conceptual model of the health research system. In this research, the so‐called conceptual model has been developed using the opinions of academic experts and appropriate operational research techniques.

The components identified in the health research system can take on different roles depending on their effectiveness or influence as well as their relationships with other components.

Management and leadership, systems and databases, financing, human resources, and research process components are located in the second quarter of the Mic Mac direct influence map. These components are the inputs of the health research system and are mainly influenced by events outside this system. Therefore, they can also be called environmental variables. Some changes in these components can completely affect the system under review, and even in some cases, defects in these components can lead to a crisis in the entire health research system. Since the research process component is located near the coordinate center, it can play a regulatory role in the research system.^26^

The components of innovation, sustainability, and culturalization are located in the third quarter of the map. According to Mic Mac maps, these variables are considered independent and have little effect on the research system. The sustainability component is near the coordinate center, just like the research process component, so it is one of the regulators of the research system.^27^, ^28^

The knowledge management and science‐producing component is a linkage component, that is, it has a high influence and dependence on the research system. This component is located in the first quarter of Mic Mac coordinates and can therefore play the role of an important and intermediate goal in the research system. Studies show that in many research systems, science production and knowledge management are the basic functions of these systems.^29^, ^30^, ^31^

The components of decision making, efficiency, satisfaction, effectiveness, equity, health promotion, and responsiveness to community needs, are among the outputs of the research system. However, paying attention to the position of these components shows that the dependency of components on health promotion and responsiveness to community needs is greater than other components of this quarter.^32^, ^33^

**Figure 2:**
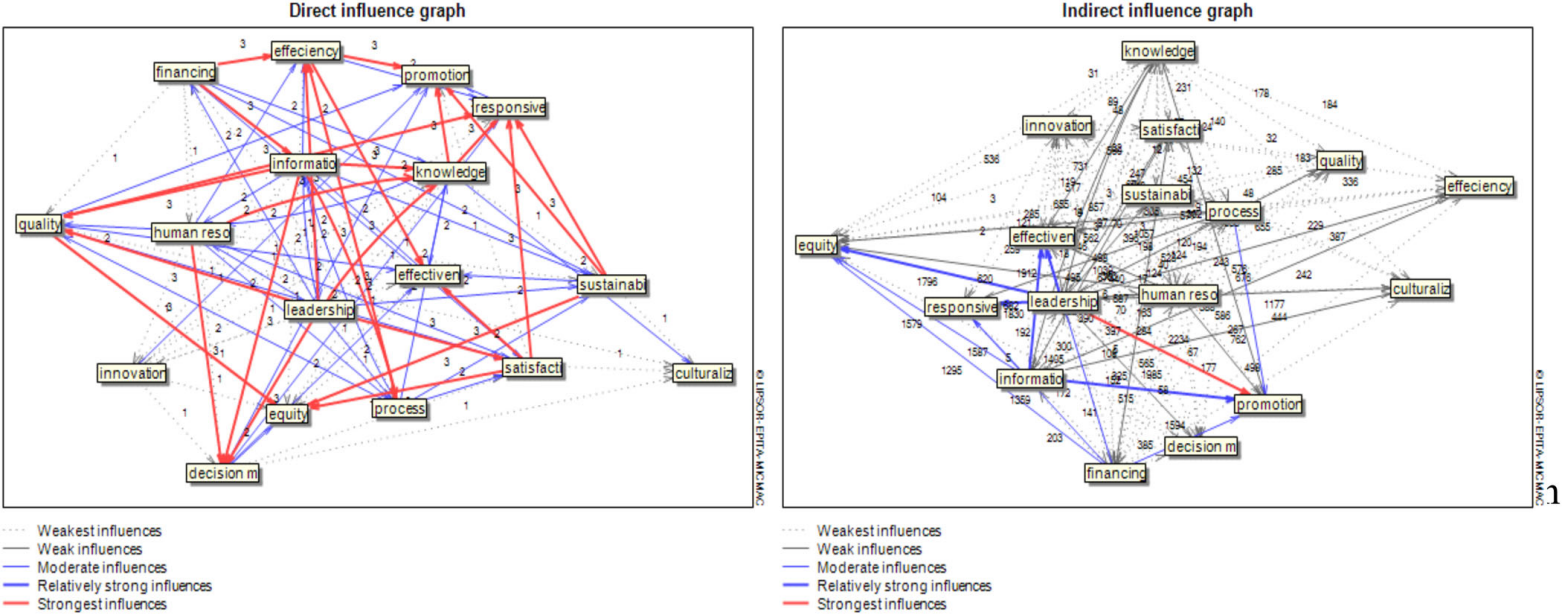
Graph of direct and indirect influence of effective components on the integrated health research system.

**Figure 3:**
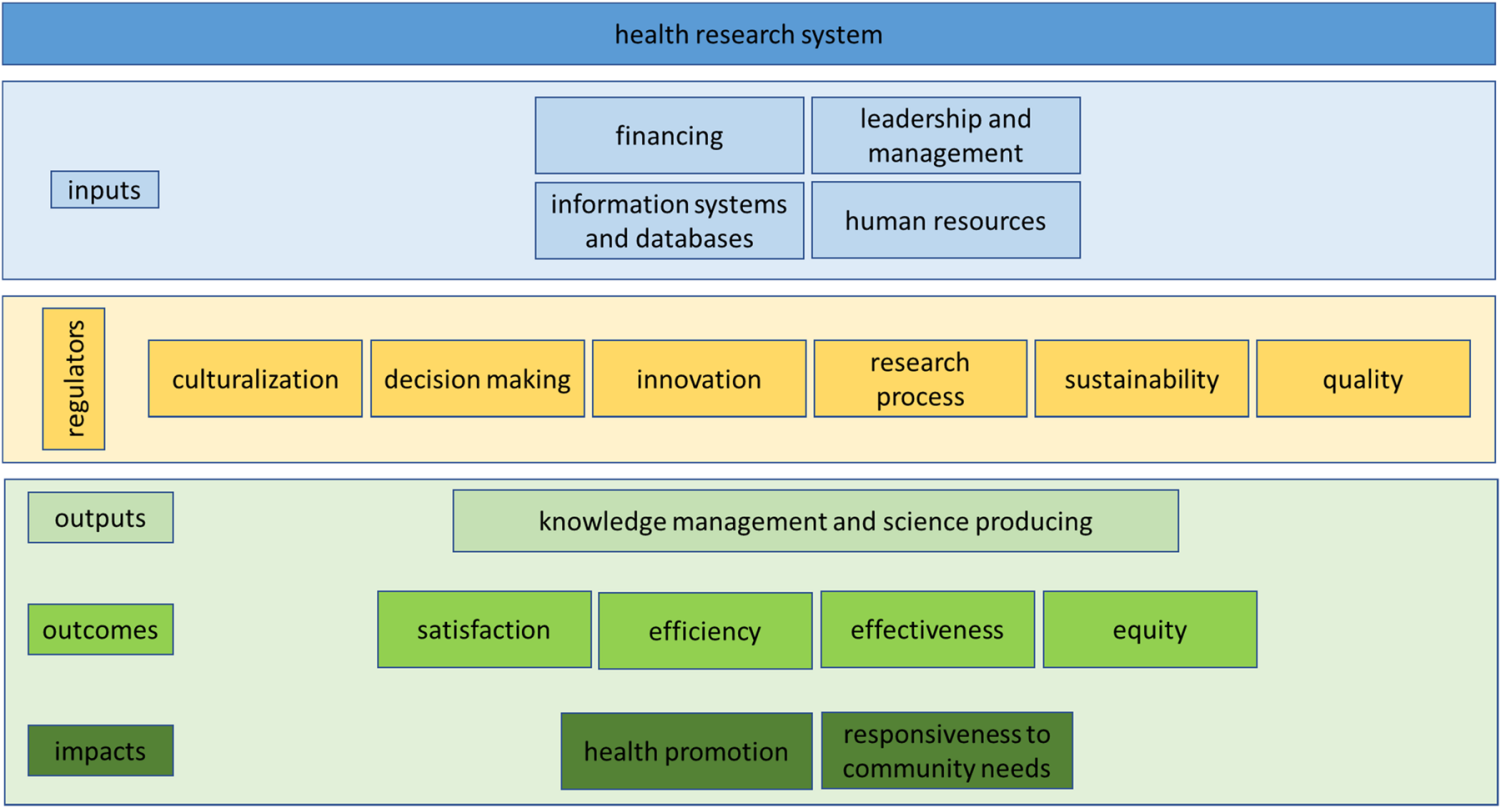
Conceptual model of integrated health research system components.

Considering that some of the studied components are located near the coordinate center and play a regulatory role, the research experts developed a conceptual model of the health research system, using the outputs of Table 1 and Figure 2. In this model, the basic concepts of the process are used. To achieve the real position of each component, direct and indirect influence numbers related to each component have been used. The model presented in Figure 3 can provide a clearer analysis of the role and position of the components.

To create a useful research system, it is necessary to provide sufficient and appropriate financial and human resources (Figure 3). Human resources are the most important input of such a system, and other inputs are formed based on the needs of this component.^34^, ^35^ As a rule, the existence of an effective database is one of the requirements of any research system, and to guide these three components, a management and leadership system is needed. The mentioned components constitute the inputs of the research system in the health system.

It is very important to pay attention to the role of regulators in this model. Components such as research process, research sustainability, and quality or innovation in research can keep research systems balanced in the health sector and ensure the survival of such systems.^36^ Also, the sustainability of such a system requires culturalization in each of the health systems.

Classifying research system outputs into three categories of outputs, outcomes, and impacts can be very helpful in policy‐making, planning, and evaluation of research systems. Science production and knowledge management are, in fact, the primary outputs of any research system.^37^ If research systems have appropriate infrastructures to create, transfer, develop, and access knowledge, it is possible to produce systematic science in those systems, and it can be hoped that this science will be used in other outputs of the health research system such as effectiveness or health promotion.^38^

## 5 CONCLUSION

In epidemic crises, health systems must focus their scientific efforts on identifying the causes of outbreak and prevalence and prevention and treatment methods of the epidemic. The COVID‐19 pandemic has challenged health systems recently, and research systems must identify and discover its causes and solutions. Combining a process model with qualitative techniques such as content analysis and quantitative methods such as Mic Mac can be beneficial in designing such systems. One of the essential inputs in such a system is the component of management and leadership, which can improve health in communities in the long run, along with other elements.

## AUTHOR CONTRIBUTIONS

**Erfan Kharazmi**: Conceptualization; data curation; formal analysis; investigation; resources; supervision; validation; visualization. **Sedigheh Ostovar**: Conceptualization; data curation; formal analysis; methodology; resources; supervision; writing—review and editing. **Milad Ahmadi Marzaleh**: Conceptualization; data curation; methodology; project administration; resources; supervision; validation; writing—original draft; writing—review and editing.

## CONFLICT OF INTEREST STATEMENT

The authors declare no conflict of interest.

## ETHICS STATEMENT

This study is derived from a research project approved with code 24176 and the ethical code IR.SUMS.NUMIMG.REC.1400.034 by Shiraz University of Medical Sciences. After obtaining the necessary permission from the Deputy of Research, the researchers presented the research samples, introduced themselves to the participants, explained the objectives of the research, and reassured them that all the recorded issues would be kept confidential. Afterward, the participants who were willing to participate in the study were selected, and they were also assured that they could withdraw from the interview process at any stage. Other ethical considerations included: (1) obtaining written consent from the experts, (2) assuring participants that the study's results would be provided to them if they so desired, (3) observing the ethical considerations in terms of confidentiality of the data, (4) acknowledging and appreciating all the people who cooperated in the research, and (5) obtaining approval from the ethics committee.

## TRANSPARENCY STATEMENT

The lead author Milad Ahmadi Marzaleh affirms that this manuscript is an honest, accurate, and transparent account of the study being reported; that no important aspects of the study have been omitted; and that any discrepancies from the study as planned (and, if relevant, registered) have been explained.

## Data Availability

Not applicable.
